# Prediction and assessment of drought effects on surface water quality using artificial neural networks: case study of Zayandehrud River, Iran

**DOI:** 10.1186/s40201-015-0227-6

**Published:** 2015-10-08

**Authors:** Hamid R. Safavi, Kian Malek Ahmadi

**Affiliations:** Department of Civil Engineering, Isfahan University of Technology, Isfahan, Iran

**Keywords:** Discharge, Drought, Temperature, Electrical conductivity, Artificial neural networks, Multi layer perceptron, Radial basis function

## Abstract

Although drought impacts on water quantity are widely recognized, the impacts on water quality are less known. The Zayandehrud River basin in the west-central part of Iran plateau witnessed an increased contamination during the recent droughts and low flows. The river has been receiving wastewater and effluents from the villages, a number of small and large industries, and irrigation drainage systems along its course. What makes the situation even worse is the drought period the river basin has been going through over the last decade. Therefore, a river quality management model is required to include the adverse effects of industrial development in the region and the destructive effects of droughts which affect the river’s water quality and its surrounding environment. Developing such a model naturally presupposes investigations into pollution effects in terms of both quality and quantity to be used in such management tools as mathematical models to predict the water quality of the river and to prevent pollution escalation in the environment.

The present study aims to investigate electrical conductivity of the Zayandehrud River as a water quality parameter and to evaluate the effect of this parameter under drought conditions. For this purpose, artificial neural networks are used as a modeling tool to derive the relationship between electrical conductivity and the hydrological parameters of the Zayandehrud River. The models used in this research include multi-layer perceptron and radial basis function. Finally, these two models are compared in terms of their performance using the time series of electrical conductivity at eight monitoring-hydrometric stations during drought periods between the years 1997–2012.

Results show that artificial neural networks can be used for modeling the relationship between electrical conductivity and hydrological parameters under drought conditions. It is further shown that radial basis function works better for the upstream stretches of the river while multi-layer perceptron is more efficient for the downstream stretches.

## Introduction

In recent decades, the available water has decreased to the extent that it barely, if at all, meets the human demands or the requirements for preserving the biological systems. Pollution and water scarcity are the two most important challenges facing most countries, especially those in arid and semi-arid regions. In this context, much attention has been focused on the physical availability of water resources at the expense of neglecting water quality which is also a main concern. Nowadays, an integrated and systematic approach to qualitative and quantitative management of water resources has gained a great significance due to the increasing components of these systems, the complex interrelationships, and their far reaching effects. For example, according to the Malaysia’s Department of Environment, many rivers experience a loss of quality, which in turn affects people’s health, the nation’s economy, and the environment [1]. The main causes of river pollution are often associated with people’s attitudes and their lack of environmental awareness. This pollution is diffused due to development along the river [2].

On the other hand, periods of drought and low flows can have dramatic effects on aquatic systems by reducing the quantity of river flows [3]. The impacts of drought conditions on river water quality may be substantial. Although the drought appeared to have significant adverse environmental effects, the actual impacts on water quality are not well understood. Typical effects are increases in total dissolved solids and their constituent ions and biochemical oxygen demand, and decreases in dissolved oxygen [4]. There have been few studies evaluating impacts of droughts and low flow rivers on water quality or aquatic systems. These studies focused on modeling and discussing the possible impacts of drought and low flows on water quality [5–14]. Most of the models developed are complex and require a significant amount of field data to support analysis.

Recently, the neural networks approach has been applied in the areas of water engineering. Artificial neural networks are able to accurately approximate complicated non-linear input–output relationships. ANN model is flexible enough to accommodate additional constraints that may arise during its application. Moreover, the ANN model can reveal hidden relationships in historical data, thus facilitating the prediction and forecasting of water quality [2, 15, 16]. Many studies have been reported on water quality modeling and prediction by using ANNs [17–23]. Hence, motivated by successful applications in modeling non-linear system behaviors, ANNs are used in the present study for modeling and prediction of surface water quality in drought or low flow conditions.

The objective of this study is to predict and simulate electrical conductivity (EC) as a water quality parameter and to assess this parameter in drought conditions for the Zayandehrud River flows in west-central Iran. In this research, the relationship between electrical conductivity and hydrological parameters of the river investigated is obtained by artificial neural networks as the modeling tool hereinafter we can estimate the relation between hydrological parameters and water quality parameter. This modeling tool consists of the multi-layer perceptron (MLP) and the radial basis function (RBF). Finally, the two models are compared with respect to their performance.

## Materials and methods

### Study area

**Fig. 1:**
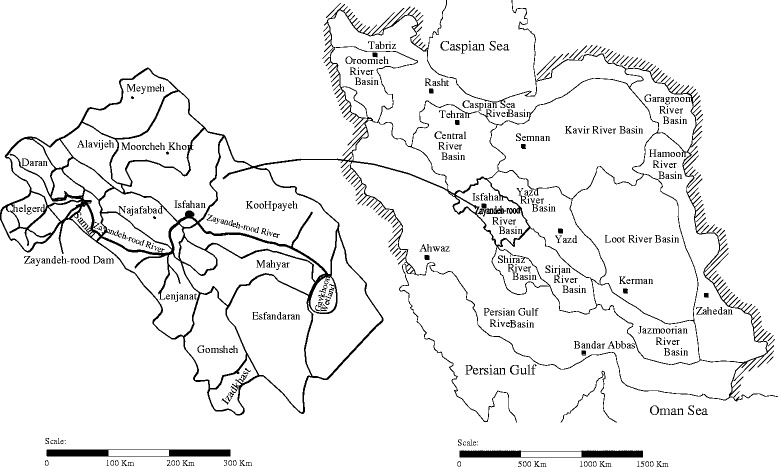
The Zayandehrud River basin in Iran

The Zayandehrud River basin covers an area of 26,917 Km^2^ located between latitudes 31^0^ 15’ and 33^0^ 45’ north and longitudes 50° 02’ and 53° 20’ east in west-central Iran (Fig. 1). The total precipitation in the basin varies between 1500 mm in the west and 50 mm in the east with an average annual value of 140 mm, which ranks the basin as a semi-arid region. The mean annual temperature in the basin is 14.5 °C with a low of −12.5 °C in January and a high of 42 °C in July. The potential annual evapotranspiration in the region is 1900 mm [24].

The Zayandehrud River is the most important river in the basin originating in the eastern slopes of the Zagross Mountain Range. The Zayandehrud storage dam with an efficient storage capacity of 1400 MCM is located 75 Km downstream the origin of the Zayandehrud River which has a natural average flow of about 900 MCM. To augment the water supply in the basin and to keep up with the increasing demand, inter-basin transfers have been implemented. Three tunnels have been constructed and are currently being operated which deliver an annual flow of 850 MCM into the basin. The flow downstream the dam supplies water for agricultural, municipal, and industrial uses. The total river length spans over a route of 350 Km to end in Gavkhooni wetland [25].

In recent decades, water has become increasingly scarce and the Zayandehrud basin has shown signs of salinization of agricultural land and increased pollution in the lower reaches of the river. While the river is subjected to multiple human impacts including water abstraction for domestic use in urban and rural areas, industrial and agricultural uses, and urban and agricultural runoff and drainage, it has also been receiving raw and treated sewages. Furthermore, the severe drought in recent years is a current phenomenon affecting water quantity and quality in the basin. Water quality generally shows a considerable spatial variability from upstream to downstream and deteriorates from Isfahan city downward the river’s course. The objective of this article is to evaluate the impact of droughts and low flows on the water quality of the Zayandehrud River.

## Methods

### Artificial neural networks

#### General concepts of artificial neural networks

An artificial neural network is created to mimic natural neural networks using computing processes. ANN models have been used to model wrapped non-linear input–output relationships in water resources management and environmental fields [26]. ANNs receive a number of inputs in the processing units which are able to communicate by sending signals to each other through a large number of weighted connections. In each network, some basic features are presented such as a set of inputs, connections within each unit, an output from each unit, an external input called bias, the rule which determines the effective input from inputs, and an activation or transfer function (usually sigmoid) which computes the correlation between the sum and the output of the unit [27].

The main idea of neural networks is that parameters can be adjusted so that the network exhibits some desired or interesting behavior. Thus, we can train the network to do a particular job by adjusting the weight or bias parameters, or perhaps the network itself will adjust these parameters to achieve some acceptable end [27]. The natural behavior of hydrological processes, and especially water quality, is appropriate for using the ANN approach. However, hydrological applications of ANN are still in their dehiscence stages [19].

#### Network training

The learning capability of ANNs is one of their interesting features. The purpose is to provide the network with a set of inputs for it to produce a certain set of outputs or at least to produce the desirable ones. The ANN processes sets of inputs and outputs in the vector phase. During periods of network learning, the weights gradually converge to desirable values. Actually, prediction error in learning a set is minimized by proper adjustment of weights. If the network learns properly, the model can produce outputs for unknown sets of inputs. There are two types of training used in ANNs: supervised and unsupervised [27, 28].

#### Multilayer perceptron neural network

**Fig. 2:**
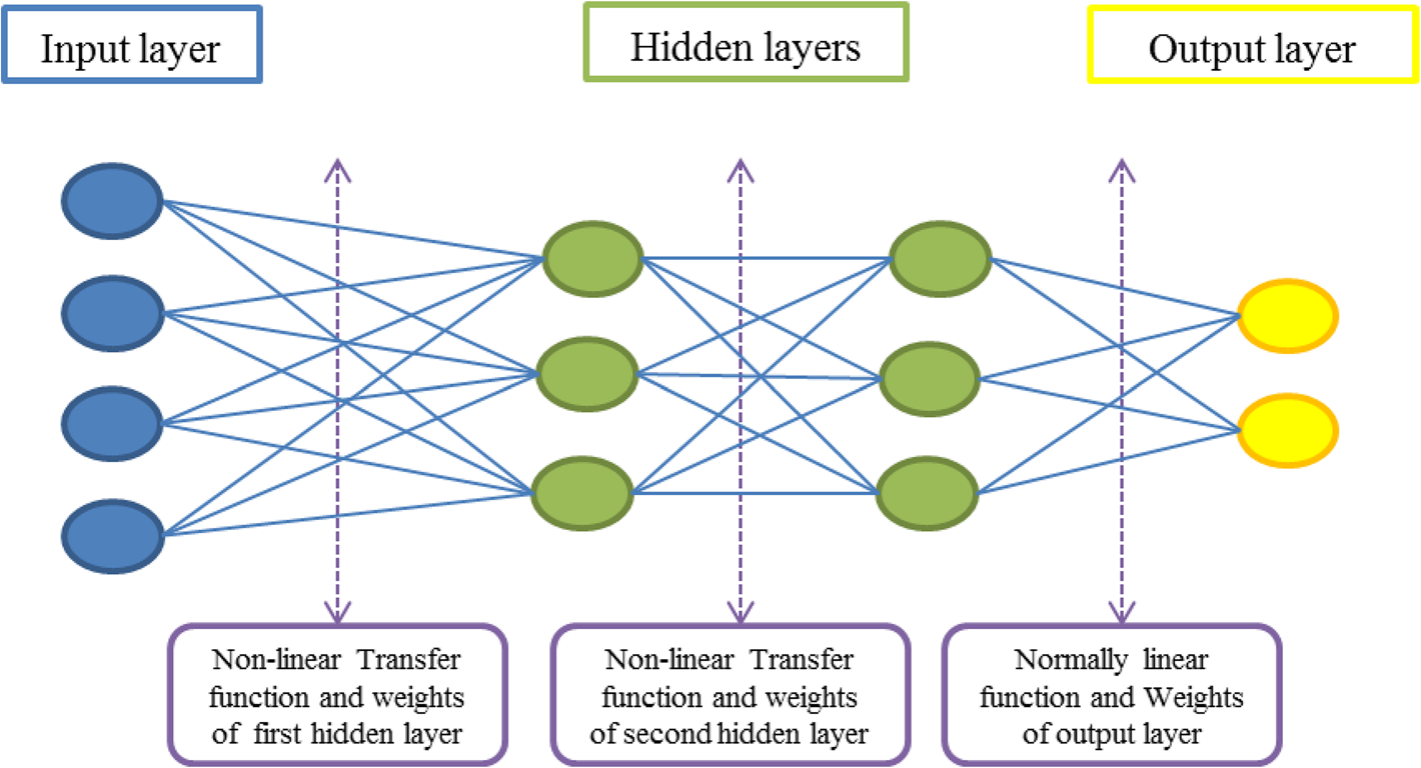
MLP-NN structure

In recent years, the feed-forward ANN, multilayer perceptron (MLP), or back-propagation network have been widely used. The MLP possesses the transfer function and different learning rules such as the delta-rule and back-propagation. The MLP is involved with function approximation and finding relationships between inputs and outputs (Fig. 2).

Two different algorithms are available that can be used for training the MLP model: 1) delta rule, and 2) back-propagation rule. In this study, back-propagation is used to construct the MLP because it is the prevalent algorithm for training MLPs (for applications of the delta rule, [28] may be consulted). Back-propagation is used to extend the delta rule and, when sets of inputs are applied to the network values of weights, biases propagate to the output unit and the mean square errors between outputs of the network and the target is computed. These values should be set to zero. Then, the weights are adjusted. Tuning the weights is a stage in which the computed errors propagate from the output layer back to the input layer. These steps are performed iteratively until errors are minimized. The errors are computed by the following equation:

1 $$ MSE=\frac{1}{N}{\displaystyle \sum_{i=1}^N}{\left(T-{Y}_i\right)}^2 $$

Where, MSE is mean square error, N is the number of observations, T is the observation value, and Yi is the prediction or output value. Back-propagation learning rule may proceed in either of two ways: 1) the pattern or case by case mode; 2) the batch mode. In the former mode, calculations are performed after each case, while in the latter, updating the calculations and weights is performed after the whole training pattern is presented [27].

#### Generalizing multilayer perceptron neural network

After the learning stage is completed, the network enters the prediction stage in which the input vector which was not presented in the learning stage is applied to the network and the corresponding outputs are predicted. The ability of a network to predict such unknown outputs is called ‘interoperability’ or ‘generalization’. One of the obstacles against the learning stage is over-fitting or over-learning of ANN on training data by which is meant the error on the training data is reduced to a minimum, but the error is still high as a result of explicitly presenting unknown data as the set of inputs so that the network is not properly generalized. One solution proposed for generalizing the network is that the network is used in appropriate dimensions. Using the network with greater dimensions may result in over-fitting. A second solution for improved generalization of the network is regularization, which will not be further discussed in the present article.

In early stopping, the data is broken down into three categories. The first is the training data set that is used for adjusting weights and for training the network. The second category consists of the validation set. During the training process, routine training is supervised. The error of the validation set should decrease as with the training set errors. When the network is on the verge of over-fitting, the validation error begins to grow and training is stopped. The third category involves the test set. This set is not employed during the training and comparing processes if diverse models are performed by this set.

#### Radial basis function neural network

The radial basis function was first developed by Broomhead and Lowe in 1988 [29]. The ordinary RBF algorithm is considered as a curve fitting operation to find the best input and output adaption and an RBF-NN gives an approximation of any input–output relationships. The constant structure of RBF consists of an input layer, a hidden layer, and an output layer. The hidden layer applies a non-linear transformation from the input space to the hidden layer. The output layer applies a linear transformation from the hidden space to the output space. The radial basis is the hidden functions. Among the several radial basis functions, the Gaussian is the one commonly used. If a Gaussian function is used, the output of each hidden layer unit then corresponds to the distance of the input from the center. This means that the transfer function of the hidden layer is Gaussian [30]. The Gaussian function takes the following form:

2 $$ \varphi \left(x,\mu \right)={e}^{- \frac{{\left\Vert x-\mu \right\Vert}^2}{2{d}^2}} $$

Where, μ is the center of the Gaussian function and d is the distance (radius) from the center of φ(x, μ) which gives a measure of the spread of the Gaussian curve.

**Fig. 3:**
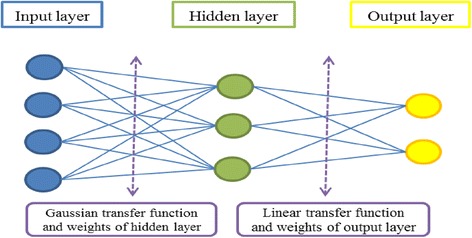
RBF-NN structure

During the training procedure, the center “μ” and the spread “d” are parameters to be determined. We can deduce from the Gaussian radial function that a hidden unit is more sensitive to data points near the center. This sensitivity can be adjusted by controlling the spread d. It must be noted that the neuron’s transfer function should cover the whole significant zone of the input space. The structure of RBF-NN is presented in Fig. 3.

Based on the type of neurons chosen from among those existing in the hidden layer, one of two methods may be employed for training the RBF-NN. The first is an exact design while the second is a more efficient design. In the first method, the numbers of hidden layer neurons are considered to be equal to the number of inputs. In the second method, one neuron is added each time to previous neurons individually till the minimum error is yielded [28]. In this research, we used the more efficient error for modeling.

#### Method of presenting input and output for training the network

It is better to present and apply input and output sets to the network in a random manner. If the data in the input file are categorized and sorted or applied to the network in a specified sequence, the network may forget what it is to learn. In fact, the network learns relationships between the input and output data but when new data are presented to the network, the error value may increase. Random presentation of data is one of the efficient routes to escape local minimization [28].

#### Network operation

Network operation is defined so as to demonstrate that the network has a reasonable response to the data which is not already stored during the training process. It is computed by three valid statistical evaluation criteria such as correlation coefficient (determination coefficient), root mean square error, and mean absolute error as expressed below:

3 $$ {R}^2=1-\frac{{\displaystyle {\sum}_{i=1}^N}{\left({O}_i-{T}_i\right)}^2}{{\displaystyle {\sum}_{i=1}^N}{\left({O}_i-{\overline{O}}_i\right)}^2} $$

4 $$ RMSE=\sqrt{\frac{\frac{1}{N-1}{\displaystyle {\sum}_{i=1}^N}{\left({O}_i-{T}_i\right)}^2}{\frac{1}{N}{\displaystyle {\sum}_{i=1}^N}{\left({T}_i\right)}^2}} $$

5 $$ MAE=\frac{1}{N}{\displaystyle {\sum}_{i=1}^N\left({O}_i-{T}_i\right)} $$

Where, O_i_ and T_i_ represent the exact or real value of the output (observation) and the predicted (test) value, respectively. N is the number of observations and Ō_i_ is the mean of the exact value.

If RMSE and MAE are close to zero, this will indicate that the prediction result is more accurate. R^2^ Anywhere close to 1 indicates that a better adoption was obtained through the exact and prediction values.

### Water quality and hydrologic data

**Fig. 4:**
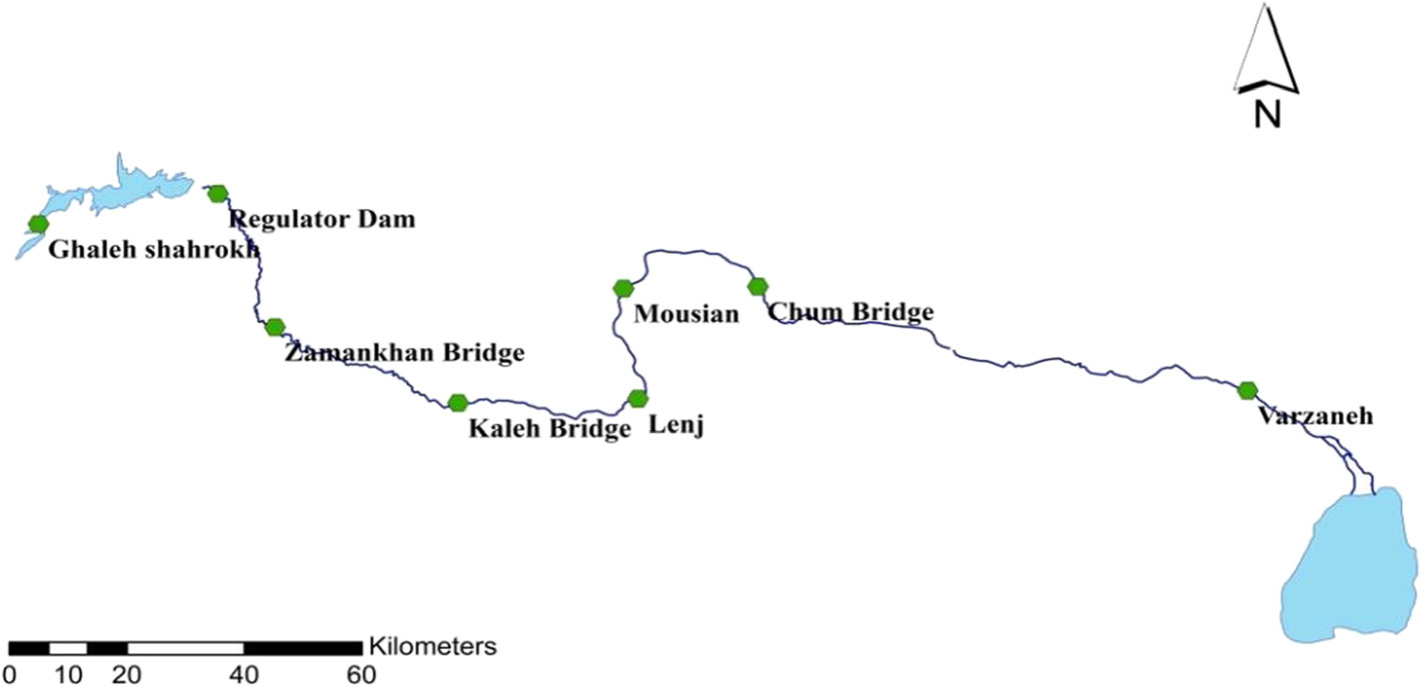
Location of hydrometric stations along the Zayandehrud River

**Fig. 5:**
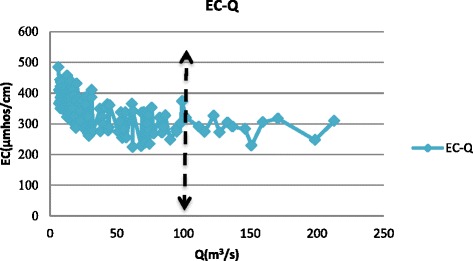
Ghaleh-shahrokh drought border

**Fig. 6:**
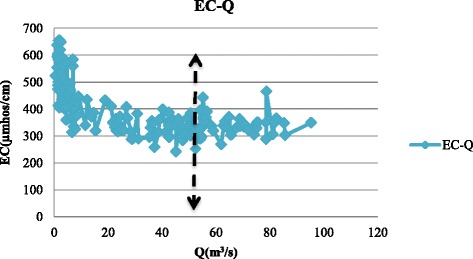
Kaleh-bridge drought border

**Fig. 7:**
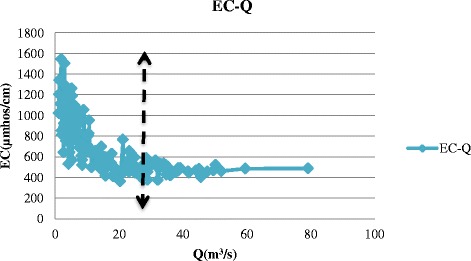
Mousian drought border

**Fig. 8:**
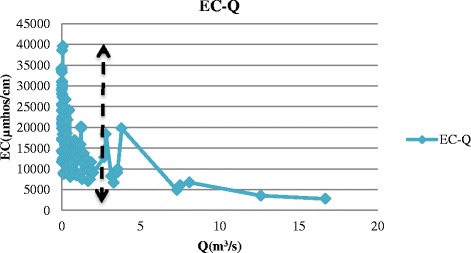
Varzaneh drought border

The data used in this study were obtained from Isfahan Regional Water Company including discharge, temperature, and electrical conductivity (EC) at the eight hydrometric stations along the Zayandehrud River between September 1997 to August 2012 which included both drought and wet years (Fig. 4). Drought or low flow threshold was determined from the discharge data from each hydrometric station. For example, drought borders for Ghaleh-shahrokh, Kaleh-bridge, Mousian, and Varzaneh stations are shown in Figs. 5, 6, 7 and 8.

Based on these Figures, the drought or low flow borders for the 8 hydrometric stations from upstream to downstream were 100, 85, 70, 50, 30, 25, 18, and 2 m^3^/s. The variations in temperature, discharge, and electrical conductivity for these eight stations are shown in Table 1.

**Table 1:** Variations at hydrometric stations for drought conditions

		Temp (°C)			Q (m^3^/s)			EC(μmhos/cm)		
No.	Station.	Max	Min	Ave	Max	Min	Ave	Max	Min	Ave
1	Ghaleh-shahrokh	22.5	−21	9.03	99	6.35	29.81	484	224	351.70
2	Regulator dam	28.5	−12	12.06	81.9	0.04	36.23	373	253	310.33
3	Zamankhan-bridge	25.5	−4.75	11.35	69.7	5.92	34.31	432	263	324.17
4	Kaleh-bridge	26.88	−1.13	12.14	49.4	0.43	16.85	655	242	418.31
5	Lenj	31.13	0.38	15.18	30	0.05	11.5	1790	407	835.65
6	Mousian	29.75	−3.75	13.04	25	1.09	9.90	1547	363	789.23
7	Chum-bridge	32.25	−1.03	15.56	17.2	0.41	6.18	1550	420	957.38
8	Varzaneh	32.7	−3.25	15.53	1.99	0.1	0.38	39600	7100	17850.4

### Modeling of water quality using neural networks

For modeling and predicting electrical conductivity (EC), we used MLP-NN and RBF-NN models. Hydrological parameters were used in the network as important factors affecting electrical conductivity to predict EC appropriately. Matlab software Ver. R2011b was used to build both networks with four input vectors. Discharge at present (t), discharge at a previous period (t-1), mean temperature at present (t), and electrical conductivity at a previous period (t-1) were fed as the sets of inputs to simulate electrical conductivity in the present time (t).

**Fig. 9:**
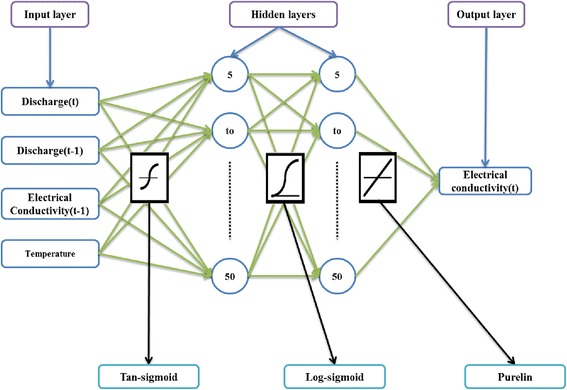
A four-layer MLP-NN for predicting EC

In the MLP-NN model, two hidden layers were used while the numbers of neurons varied from five to fifty for each station (Fig. 9). Because there is no general rule for determining the properties of hidden layers and the neurons, trial-and-error procedures recommended by many researchers [2] were used to construct the hidden layer and the neurons. The number of hidden layer neurons significantly influences the performance of a network: if the number is small, the network may not achieve the acceptable level of accuracy, but if there are too many, training may be lengthy and the model may over-fit the data. Two loops were used to build the first and the second hidden layers and the efficient results obtained were stored at each point in time. These results were procured based on the assumption that the network should not run into over-fitting and that the error should have decreased by increasing number of neurons. MLP-NN was trained by the back-propagation rule and the Levenberg-Marquardt optimization of weights and bias values. The transfer function for the first hidden layer was tangent-sigmoid while it was log-sigmoid for the second. Based on the data sets, 70 % of the data sets were used for training and 30 % for both testing and validation of the network.

**Fig. 10:**
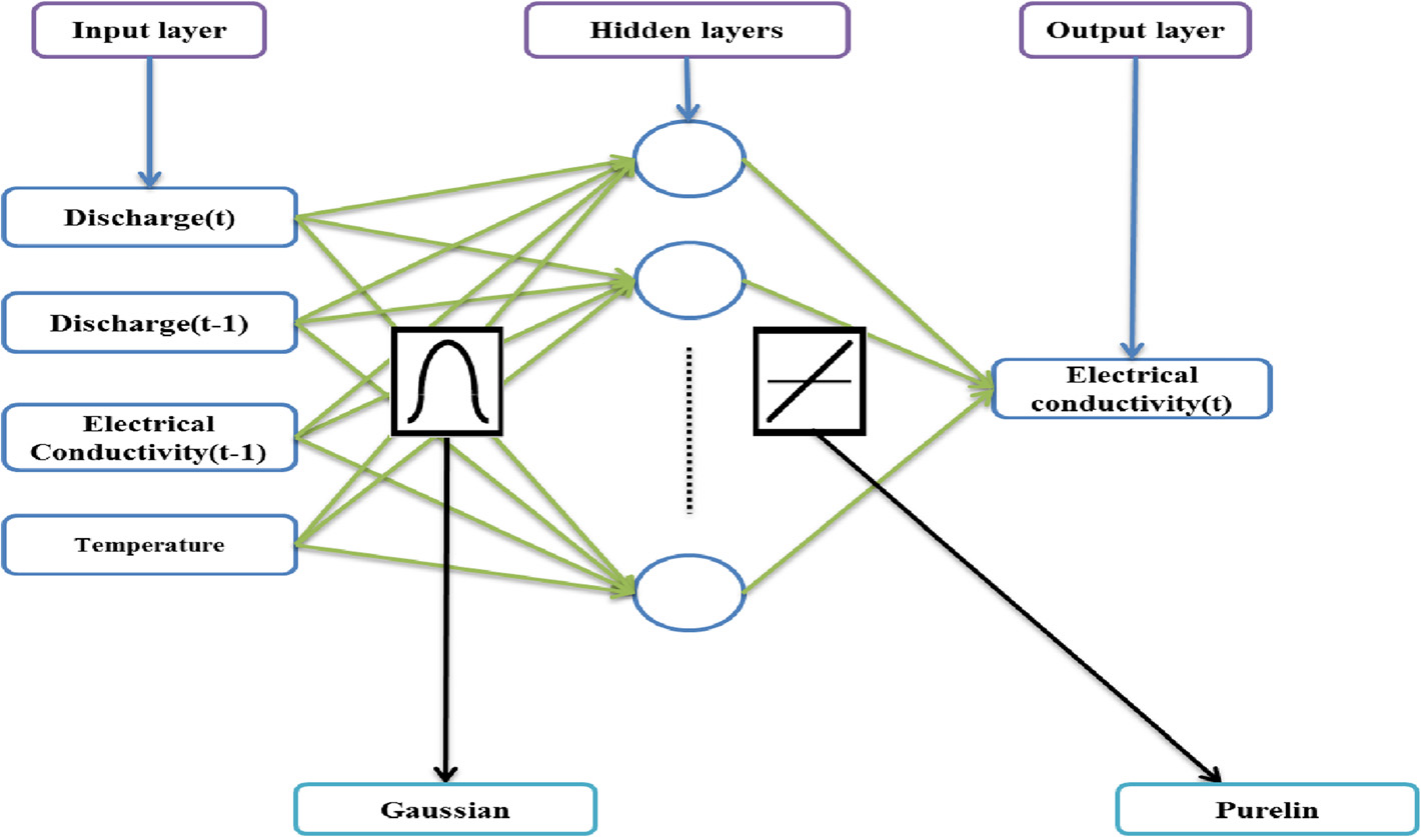
RBF-NN model for predicting EC

The RBF-NN has a constant structure. The ability of the RBF-NN model to achieve the target depends to the predefined internal parameters such as the number of neurons and the spread. The number of neurons defines the contribution of each input parameter to the desired output while the spread controls the adaptive changes that the RBF-NN makes to the neurons. During training, optimization of RBF-NN parameters is an important stage for appropriate mapping. This optimization is performed by the efficient design method and the trail-and-error process for determination of spread and neurons of the hidden layer (Fig. 10). Based on the data sets, 90 % of the data sets were used for training and 10 % for testing the network.

The performances of the models are evaluated using determination coefficient (R^2^), root mean square error (RMSE), and mean absolute error (MAE).

## Results and discussion

**Fig. 11:**
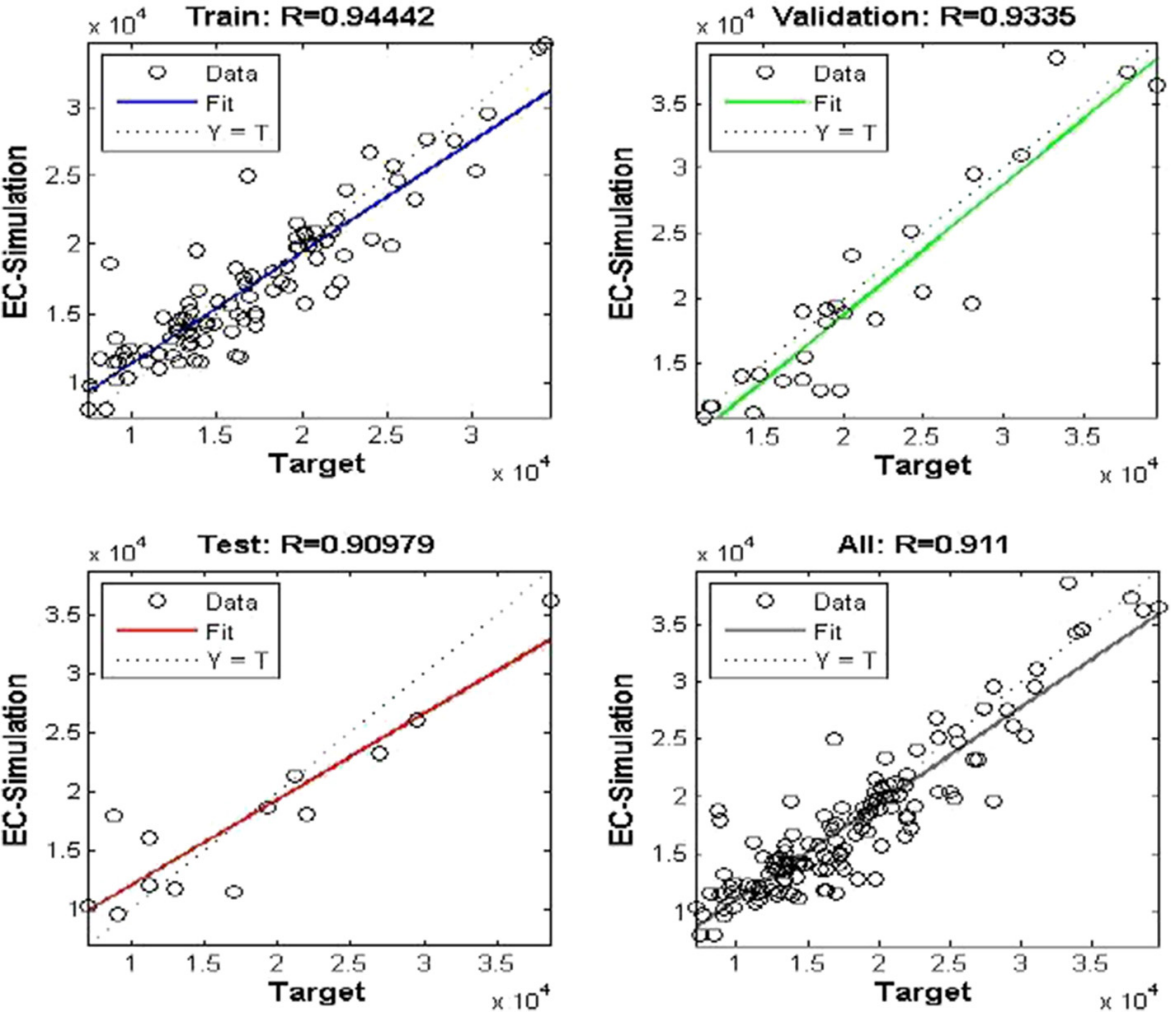
MLP-NN correlation result for train, validation, and test group and all data for Varzaneh station

**Fig. 12:**
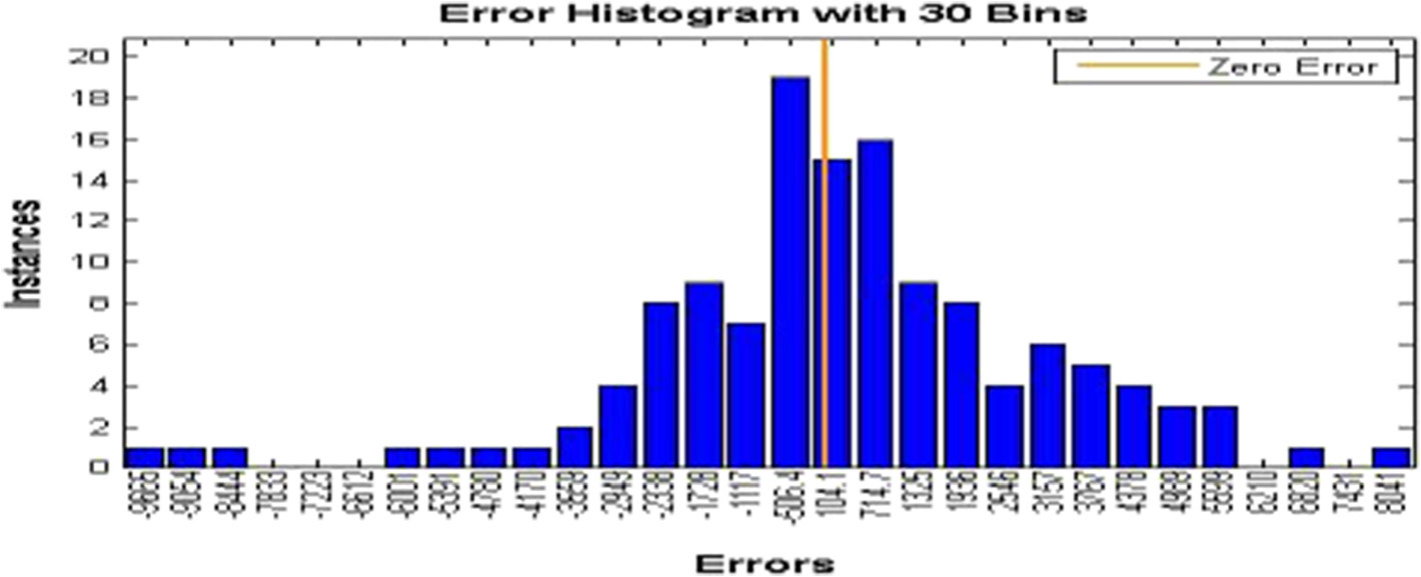
MLP error histogram for Varzaneh station

Sixteen architectures (MLP and RBF) were developed to simulate electrical conductivity in drought or low flow conditions. All the networks achieved the efficient MSE (mean square error) during training. Choosing the proper inputs when creating the models has a great impact on their performance. After training the proposed model, the next step is to test the model with the test data sets. In the MLP model, the network was optimized by ten neurons in the first hidden layer and four neurons in the second one. The values of RMSE and R^2^ for the training set, the validation set, and the test set were (2541.63, 0.8918), (3242.06, 0.8714), and (3893.45, 0.8275), respectively, in Varzaneh station (Fig. 11). Also, the error histogram for this station shows the maximum absolute error prediction was 9665 while its minimum was 104.1 μmhos/cm. For brevity, the MLP-NNs features and performances for each of the eight stations are summarized and only the correlation diagram and the error histogram for Varzaneh station are presented (Fig. 12).

**Fig. 13:**
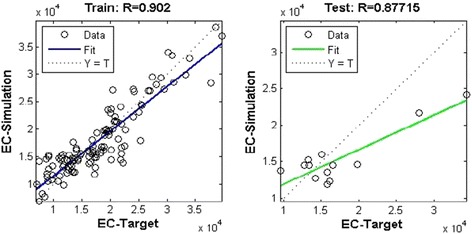
RBF-NN correlation results for train and test for Varzaneh station

In the RBF model, the network was optimized by 45 neurons in the hidden layer (or radial function) and 955 as the spread, whose RMSE and R^2^ for the training set and the test set were (2988.51, 0.8136) and (4267.78, 0.7693), respectively, in Varzaneh station (Fig. 13).

**Fig. 14:**
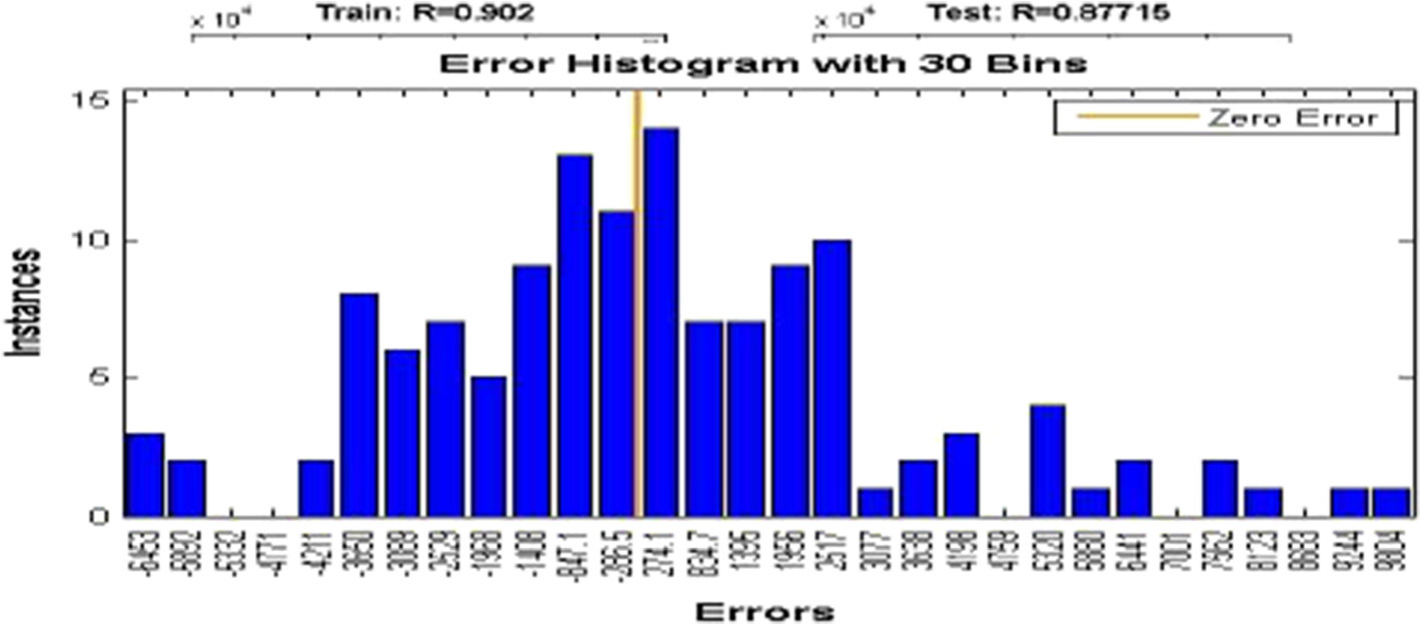
RBF error histogram for Varzaneh station

Also the error histogram is presented for this station which shows that the maximum absolute error prediction was 9804 and its minimum was 274.1 μmhos/cm. Similar to MLP-NN, the MLP-NNs features and performances for each of the eight stations are summarized and only the correlation diagram and the error histogram for Varzaneh station are presented in Fig. 14.

It is clear that, with respect to their training performance, the models (MLP-NN and RBF-NN) developed were capable of imitating the electrical conductivity accurately with relatively low-error for all the samples provided. The results also demonstrate that MLP-NN and RBF-NN were able to perceive the input–output mapping in the historical data and to interpolate the unseen pattern for better prediction in drought conditions (Tables 2 and 3).

**Table 2:** Summary results for MLP-NN for simulating EC

		Transfer function			Optimized number of neurons		Performance							Errors (μmhos/cm)	
Station	Total number of data	First hidden layer	Second hidden layer	Output layer	First hidden layer	Second hidden layer	Train		Validation		Test		MAE	Max absolute error prediction	Min absolute error prediction
							RMSE	R^2^	RMSE	R^2^	RMSE	R^2^			
Ghaleh-shahrokh	141	Tan-sigmoid	Log-sigmoid	Purelin	10	4	22.08	0.82	22.15	0.82	28.66	0.75	22.83	65.04	1.23
Regulator dam	145				17	8	16.06	0.62	16.14	0.57	14.43	0.67	11.13	64.29	1.18
Zamankhan-bridge	129				5	4	17.50	0.68	19.02	0.60	20.40	0.69	16.97	58.55	0.60
Kaleh-bridge	106				17	10	30.26	0.91	33.77	0.80	60.12	0.79	47.79	96.31	1.27
Lenj	110				13	13	98.20	0.90	116.33	0.90	242.89	0.83	202.82	420.00	6.82
Mousian	105				7	5	88.07	0.89	93.24	0.88	117.26	0.85	84.15	327.80	7.87
Chum-bridge	105				8	8	103.38	0.88	109.98	0.82	145.46	0.81	110.70	281.10	0.13
Varzaneh	131				8	9	2541.63	0.89	3242.06	0.87	3893.45	0.83	3046.60	9665.00	506.40

**Table 3:** Summary results for RBF-NN for simulating EC

		Transfer function		Optimized number of neurons and spread		Performance					Errors (μmhos/cm)	
Station	Total number of data	First hidden layer	Output layer	Hidden layer	Spread	Train		Test		MAE	Max absolute value of error prediction	Min absolute value of error prediction
						RMSE	R^2^	RMSE	R^2^			
Ghaleh-shahrokh	141	Gaussian	Purelin	47	16.6	21.49	0.83	27.43	0.81	23.20	71.52	0.89
Regulator dam	145			60	21	14.86	0.65	15.82	0.76	10.85	50.85	0.35
Zamankhan-bridge	129			36	37	16.66	0.71	20.89	0.69	17.28	42.8	0.26
Kaleh-bridge	106			43	17	33.82	0.89	28.77	0.88	23.03	105.3	1.69
Lenj	110			44	99	99.35	0.90	125.15	0.80	98.43	423.10	5.22
Mousian	105			22	47.6	94.75	0.87	120.88	0.82	92.98	269.70	3.49
Chum-bridge	105			41	19	122.42	0.82	245.08	0.90	211.13	379.90	2.51
Varzaneh	131			45	955	2988.51	0.81	4267.78	0.77	3466.10	9804	274.1

**Fig. 15:**
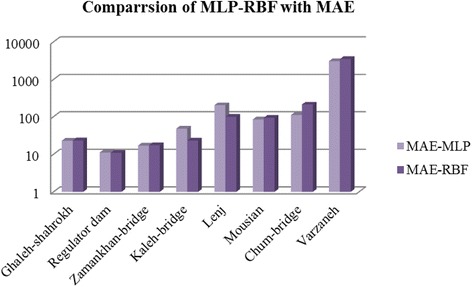
Comparison of MLP and RBF capabilities with MAE for EC simulating for all the stations

Based on the same results, if MAE is considered as a performance criterion, its network efficiency improved in Ghaleh-shahrokh, Regulator dam, Kaleh-bridge, and Lenj compared to the MLP-NN. Fig. 15 shows this comparison in the logarithmic scale represented in vertical axes. In Lenj station, MAE is observed to rise for both models while the electrical conductivity values in neighboring stations such as Kaleh-bridge and Mousian are close to that of Lenj station, indicating that electrical conductivity may depend on another parameter in the input.

For a more detailed analysis of the efficiency of the proposed model, we used a performance indicator known as the prediction error enhancement rate (PEER), proposed by [31], which is expressed as follows:

6 $$ PEER=-\frac{\left(P{E}_{Max-RBF}-P{E}_{Max-MLP}\right)}{P{E}_{Max-MLP}}\times 100 $$

Where, PE_Max-RBF_ and PE_Max-MLP_ are the maximum prediction errors for RBF-NN and MLP-NN defined by:

7 $$ P{E}_{Max}= \max \left(\frac{Y_o-{Y}_p}{Y_o}\right) $$

Where, Y_o_ represents the observed values and Y_P_ designates the predicted ones. This equation originates from a simple proportion that is commonly used for comparing two cases.

**Fig. 16:**
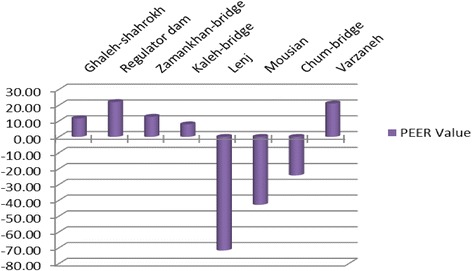
PEER results for all the stations

If PEER is greater than zero, RBF-NN is then more efficient than MLP-NN. This indicator can analyze and examine the ability of the proposed model to minimize the prediction error. Equation 6 is adapted to present the efficiency of the RBF-NN model compared to MLP-NN. According to PEER values, RBF-NN shows greater improvements in Lenj, Mousian, and Chum-bridge stations over the MLP-NN. These improvements range from 7.91 to 22.03 %, but only in Lenj station, the MLP-NN is more efficient by about 70 %. However, both these models are generally usable since they both have low errors (Table 4 and Fig. 16).

**Table 4:** Summary of PEER results for MLP-RBF comparison

Station	PE_Max-MLP_	PE_Max-RBF_	PEER
Ghaleh-shahrokh	0.2597	0.2294	11.67
Regulator dam	0.1829	0.1426	22.03
Zamankhan-bridge	0.192	0.1676	12.71
Kaleh-bridge	0.2641	0.2432	7.91
Lenj	0.491	0.843	−71.69
Mousian	0.2998	0.4281	−42.80
Chum-bridg	0.4431	0.5503	−24.19
Varzaneh	1.1394	0.899	21.10

## Conclusion

The low accuracy of classical methods and approaches such as linear regression for modeling environmental conditions and water quality, as well as the nonlinear nature of water quality problems for planning proper management systems have been discussed in numerous researches. A proper management plan is a comprehensive plan which has sufficient valence and reliability both in scientific terms and in empirical or industrial applications. ANN or the black-box model is a new technique for modeling water quality problems. It can accurately model problems involving water quality and hydrological processes provided that sufficient experimental data are available. It is also capable of discovering non-linear relations between hydrological and water quality parameters.

In this study, two different ANN models, namely the MLP and the RBF, were used to simulate and predict electrical conductivity in drought or low-flow conditions. Both networks were then compared with respect to their performance. It was found that electrical conductivity is associated with major water quality parameters and further that it is intensely depends on changes in discharge to the extent that the changes can be used as a proper water quality indicator. Significant changes in EC indicate abrupt changes in discharge or introduction of pollutants into the river. Obviously, river discharge is one of the parameters affected by hydrological droughts. Water from the Zayandehrud River is released from a regulating dam; discharge is, therefore, regulated at the downstream stations. When upstream discharge is low, a water deficit or drought conditions accrue, whereby evaporation is increased and the water stored in the dam reservoir declines. It is observed that EC increases severely at the last station near Gavkhuni Wetland where enormous biological disasters have been observed to occur which indicate the enormous agricultural activities upstream the Gavkhuni Wetland.

In this study, drought borders were determined and employed in the MLP and RBF neural networks. The results showed that when MAE is used as a criterion for comparing the networks in terms of their performances, the RBF-NN was found to outperform MLP-NN. However, based on the same criterion, both MLP-NN and RBF-NN were found to be equally reliable. According to the prediction error enhancement rate used as a criterion, the MLP-NN was found to be more efficient than the RBF-NN at Lenj, Mousian, and Chum-bridge stations. Obviously, these two criteria provided better results for MLP-NN at Lenj station. Nevertheless, both networks could be used for accurately modeling the situation at each station. Other decision making methods are suggested for investigation to validate the results obtained. Also, these neural network structures can be used as the basis for predicting and simulating water quality in diverse hydrological conditions, and for improving management approaches in river basins.

## References

[1] Department of Environment (DOE). Water quality management in Malaysia. Kuala Lumpur, Malaysia: Federal Goverment Administrative Centre; 2003.

[2] Najah A, El-Shafie A, Karim OA, El-Shafie Amr H (2013). Application of artificial neural networks for water quality prediction. Neural Comput & Applic.

[3] Tallaksen LM, Madsen H, Clausen B (1997). On the definition and modelling of stream flow drought duration and deficit volume. Hydrol Sci J.

[4] Bruce CC, Robinson DP (1987). Some effects of the 1982–83 drought on water quality and macro-invertebrate fauna in the Loer La Trobe River, Victoria. Aust J Mar Fresh Wat Res.

[5] Attrill MJ, Power M (2000). Modeling the effect of drought on estuarine water quality. Water Res.

[6] Caruso BS (2002). Temporal and spatial patterns of extreme low flows and effects on stream ecosystems in Otago, New Zealand. J Hydrology.

[7] Clair TA, Ehrman JM (1996). Variations in discharge and dissolved organic carbon and nitrogen export from terrestrial basins with changes in climate: a neural network approach. Limnol Oceanogr.

[8] Hrdinka T, Novicky O, Hanslik E, Rieder M (2012). Possible impacts of floods and droughts on water quality. J Hydro Environ Res.

[9] Murdoch PS, Baron JS, Miller TL (2000). Potential effects of climate change on surface-water quality in North America. J Am Water Resour Assoc.

[10] Nouri J, Mirbagheri SA, Farrikhian F, Jaafarzadeh N, Alesheikh AA (2010). Water quality variability and eutrophic state in wet and dry years in wetlands of the semiarid and arid regions. Environ Earth Sci.

[11] Schindler DW (1997). Widespread effects of climate warming on freshwater ecosystems in North America. Hydrologic Processes.

[12] Sprague LA (2005). Drought effects on water quality in the South Platte River Basin, Colorado. J Am Water Resour Assoc.

[13] van Vliet MTH, Zwolsman JJG (2008). Impact of summer droughts on the water quality of the Meuse River. J Hydrol.

[14] Zielinski P, Gorniak A, Krzysztof Piekarski M (2009). The effect of hydrological drought on chemical quality of water and dissolved organic carbon concentrations in Lowland Rivers. Polish J Ecol.

[15] Nasir MFM, Abdul Zali M, Juahir H, Hussain H, Zain SM, Ramli M (2012). Application of receptor models on water quality data in source apportionment in Kuantan River Basin. J Environ Health Sci Eng.

[16] Zare AH (2012). Evaluation of multivariate linear regression and artificial neural networks in prediction of water quality parameters. J Environ Health Sci Eng.

[17] Fu Y, Zhao Y, Zhang Y, Guo T, He Z, Chen J (2013). GIS and ANN-based spatial prediction of DOC in river networks: a case study in Dongjiang, Southern China. Environ Earth Sci.

[18] Ha H, Stenstrom MK (2003). Identification of land use with water quality data in stormwater using a neural network. Water Res.

[19] Maier HR, Dandy GC (2000). Neural networks for the prediction and forecasting of water resources variables: a review of modelling issues and applications. Environ Model Softw.

[20] Ogleni N, Topal B (2011). Water quality assessment of the Mudurnu River, Turkey. Using Biotic Indices. Water Resour Manag.

[21] Rooki R, Doulati Ardejani F, Aryafar A, Bani AA (2011). Prediction of heavy metals in acid mine drainage using artificial neural network from the Shur River of the Sarcheshmeh porphyry copper mine, Southeast Iran. Environ Earth Sci.

[22] Verma AK, Singh TN (2013). Prediction of water quality from simple field parameters. Environ Earth Sci.

[23] Zhang Y, Pulliainen J, Koponen S, Hallikainen M (2002). Application of an empirical neural network to surface water quality estimation in the Gulf of Finland using combined optical data and microwave data. Remote Sens Environ.

[24] Safavi HR, Khoshoei Esfahani M, Zamani AR (2014). Integrated index for assessment of vulnerability to drought, case study: Zayandehrud River basin, Iran. Water Resour Manag.

[25] Safavi HR, Chakraei I, Kabiri-Samani A, Golmohammadi MH (2013). Optimal reservoir operation based on conjunctive use of surface water and groundwater using neuro-fuzzy systems. Water Resour Manag.

[26] Haykin S (1999). Neural networks: Comprehensive foundation.

[27] Farmaki EG, Thomaidis NS, Constantinos EE (2010). Artificial neural networks in water analysis; Theory and applications. Int J Environ An Ch.

[28] Hagan MT, Demuth HB, Beale MH (1996). Neural network design, MA.

[29] Broomhead DS, Lowe D (1988). Multivariate functional interpolation and adaptive networks. Complex Systems.

[30] Bishop CM. Neural networks for pattern recognition. Oxford University Press. 1996

[31] El-Shafie A, Jaafer O, Akrami SA (2011). Adaptive neuro-fuzzy inference system based model for rainfall forecasting in Klang River, Malaysia. Int J Phys Sci.

